# A Portable Hip Arthroscopy Simulator Demonstrates Good Face and Content Validity with Incomplete Construct Validity

**DOI:** 10.1016/j.asmr.2021.05.009

**Published:** 2021-07-17

**Authors:** Aoife Feeley, Luke Turley, Eoin Sheehan, Khalid Merghani

**Affiliations:** a Department of Trauma and Orthopaedic Surgery, Tullamore Hospital, Arden, Road, Tullamore, Co. Offaly, Ireland; b Graduate Entry Medical School, University of Limerick, Castletroy, Limerick, Ireland

## Abstract

**Purpose:**

We evaluate the face, content, and construct validity of a portable hip arthroscopy module in a regional orthopaedic unit.

**Methods:**

Participants were recruited from a regional orthopaedic centre, and categorized into novice (0 arthroscopies), intermediate (1-29 arthroscopies), and expert (>50 arthroscopies) groups based on reported experience in arthroscopy. Face and content validity was evaluated by feedback from users immediately following completion of modules. Objective measurements, including time taken and subjective measurements consisting of simulation software metrics including, cam lesion locations attempts, scope strikes on bone, healthy bone burred, and cam lesion burred. Scores achieved by experts were recorded, and the median score was set at the level at which proficiency was demonstrated. Participant feedback on perceived educational use was collected following completion.

**Results:**

In total, 20 participant results were included for analysis. Good face and content validity was expressed by participants with previous arthroscopic experience. Number of scope strikes within the simulator-derived metrics accurately discerned between levels of experience. Novices had a mean of 5 strikes per attempt (SD ±5), intermediates a mean of 5.8 strikes (SD ± 4.1). There was a significant difference between expert and novice groups (*P* = .01), and expert and intermediate groups (*P* = .002). No significant difference between overall performance scores achieved by participants in expert, intermediate, and novice groups (62% ±19vs 55% ±22 vs 50% ±23,*P* = .15). This demonstrates incomplete construct validity of the simulator software-derived metrics.

**Conclusions:**

This hip arthroscopy simulator demonstrated acceptable face and content validity, with incomplete construct validity of simulator software metrics. Participants reported high levels of satisfaction with the module, highlighting that the addition of haptic feedback would be beneficial to improve procedural steps. Incorporation of tactile feedback to the modulator components would likely enable the software to accurately delineate between levels of experience.

**Clinical Relevance:**

This study demonstrates good face and content validity. The addition of haptic feedback in a hip arthroscopy simulator may improve learning.

## Introduction

Hip arthroscopy is a procedure growing in popularity, with a multitude of indications, including intra-articular and periarticular conditions. Its use is associated with a steep learning curve,^2^^,^^3^ with a wide range of cases reported to achieve proficiency, with Hoppe et al.^1^ finding a plateau in the learning curve after 30 cases. Complications associated with hip arthroscopy are numerous, including avascular necrosis of the femoral head,^4^ femoral neck fracture, and articular damage,^5^ with these structures at increased risk during the operator’s early exposure to the procedure.^1^

With the introduction of the European working time directive^6^ and shorter training schemes, surgical trainees face challenges, including increasingly complex surgical procedures^7^ with reduced operative training time. With a high rate of newly qualified surgeons reporting insufficient experience to operate independently,^8^^,^^9^ novel methods are required to counteract the training shortcomings encountered by surgical trainees. Virtual reality simulation has been reported to accelerate the practical skills learning process in a broad range of surgical specialties.^10^^,^^11^ Arthroscopy is a technically difficult surgical procedure, in part, because of the two-dimensional visual feedback while working within a 3-dimensional (3-D) field. The use of virtual reality can help users adapt to this feedback system.^12^

Although conventional virtual reality simulation is cumbersome and not easily portable, the potential advantages of mobile simulation trainers are innumerable, with remote training allowing trainees to prevent surgical skill decay during reduced access to the clinical environment.^13^ Precision OS (Vancouver, Canada) is a novel Orthopaedic Virtual Reality surgical simulator^11^ with a cam lesion hip arthroscopy module available, with a headset and controllers creating a 3-D simulated arthroscopic environment. The aim of this study was to evaluate the face, content, and construct validity of a portable hip arthroscopy module in a regional orthopaedic unit.

The authors hypothesized the Precision OS Virtual reality hip arthroscopy simulator contained sufficient face, content, and construct validity to be an acceptable adjunctive training tool to users, while being able to differentiate between levels of arthroscopic experience.

## Methods

### Study Population

This was a prospective comparative study in a regional trauma orthopaedic unit. Institutional Review Board approval was waived in keeping with regulatory guidelines.^14^ We recruited 20 volunteers and categorized participants based on self-reported experience, into three groups of novice, intermediate, and expert cohorts over an 8-week period: November 2020-January 2021. A minimum number of participants was calculated from previous validation studies evaluating arthroscopy.^15^ Written informed consent was obtained from each volunteer. The novice group contained medical students, postgraduate year 1 doctors, and core specialty trainees, with no previous experience with hip arthroscopy. The intermediate group consisted of surgical trainees who had performed between 1 and 29 hip arthroscopy procedures as the primary operator. Advanced users were defined as participants with greater than 50 hip arthroscopy procedures as a primary operator. Groups were defined in line with reported learning curves associated with hip arthroscopy.^1^ Inclusion criteria consisted of medical students on site, orthopaedics trainees, nonsurgical doctors, and consultant orthopaedic surgeons. Exclusion criteria included volunteers who were unable to complete the simulated task. This was to ensure a complete set of data was available for each participant for analysis.

### Simulator Task

Precision OS (Vancouver, Canada) is a high-fidelity Virtual Reality simulation platform focused on orthopaedics, with bimanual control and haptic feedback available in the hand -held devices, to allow virtual tools and structures to be “felt” during simulation. The simulator consists of one portable headset and two wireless handheld lightweight controllers.

Five steps are involved with the hip arthroscopy module: 1) limb traction; 2+3) greater trochanter location; 4, cam lesion location and cannula insertion; and 5) arthroscopy.

Participants were asked to place the limb under traction, palpate the greater trochanter, mark this, and using radiographs locate the cam lesion. Following this they were asked to insert the cannula within the capsule and direct its tip to the cam lesion under radiological guidance. Scope portals were then inserted with assistance from the simulator module. The participants were asked to insert the camera into the appropriate portal and sufficiently visualize the lesion arthroscopically. To complete the module, the participants were required to insert to burr and remove the cam lesion with adequate arthroscopic camera views to reduce risk of damage to healthy bone. Once participants were satisfied with the amount of cam lesion burred, they removed the instruments from the hip to complete the module.

### Simulation-Derived Metrics

With the expert group proficient at hip arthroscopy, their median composite score was calculated using the simulator software was used to determine the level at which participant proficiency was demonstrated. This study tested the null hypothesis; if no significant difference in the individual or composite scores was noted between the expert and intermediate or novice groups, this would demonstrate the simulator could not differentiate between levels of experience, thus lacking construct validity.

### Data Collection

Participants were oriented and supervised by one instructor. Instructions were given before initiation of the task, and participants were given the opportunity to familiarize themselves with the simulator prior to the procedure. Participants were asked to perform the hip arthroscopy module twice.

Measured objective and subjective simulator-derived novel metrics were collected from both attempts, for intraparticipant and interparticipant comparisons. Objective metrics included were time taken to completion and number of radiographs taken. Novel metrics collected included the precision score calculated, number of attempts taken to identify the cam lesion with the cannula under radiological guidance, number of scope strikes on the bone, amount of both healthy bone and cam lesion burred. These outcome measures were recorded by the simulator software, with metrics provided for each. The precision score is the composite score, which gives equal weight to each of the other 4 metrics computed by the simulator. The expert group’s scores were recorded and set as the standard at which proficiency was demonstrated. Correlation between the groups’ performance on the calculated metrics was then assessed.

Baseline data were collected from participants, including previous virtual reality (VR) simulator experience, video game use, and handedness. Verbal feedback on realism, satisfaction, and its use as a training tool for surgical education and training was also collected following completion of the simulated module.

### Statistical Analysis

Statistical analysis was carried out by an independent reviewer using Statistical Package for the Social Sciences version 26 (SPSS, Chicago, IL). Kruskal-Wallis test was used to assess the difference across the three groups, with the Mann Whitney *U*-test used to evaluate difference between individual groups’ performance. *T*-test calculations were used to assess the time to completion between groups. Inter-rater reliability was assessed via intraclass correlations collected. The relationship between previous VR experience and performance scores were assessed using two-way ANOVA. A *P* value <.05 was taken as level of significance in all statistical analyses.

## Results

The study participants’ demographics are available in Table 1. Nine participants were categorized as novices after reporting no prior arthroscopic experience, 9 as intermediates, and 2 experts were recruited who had >100 hip arthroscopic cases each.

**Table 1:** Participant Characteristics

Category	Number	Mean Age (Years)	Sex	Number of Hip Arthroscopies	Other Arthroscopy Experience	Handedness	Gaming Experience	Previous VR Experience
Novice	9	25 ± 4	M 44% (4/9)	<1 ± 0	<1 ± 0	R 88.8% (8/9)	11.2% (1/9)	33.3% (3/9)
Intermediate	9	32 ± 5	M 66% (6/9)	5 ± 3	20 ± 4	R 100% (9/9)	22.4% (2/9)	11.2% (1/9)
Expert	2	40 ± 2	M 100% (2/2)	>100 ± 23	>200 ± 48	R 100% (2/2)	0%	0%
Significance		*P* = .01∗	*P* = .12	*P* < .001∗	*P* < .001	*P* = .48	*P* = .47	*P* = .26

∗ Denoting significant difference.

### Face Validity

Good face validity was reported by all participants with previous experience in the procedure using a 5-point Likert scale. 100% of participants reported very acceptable or moderately acceptable levels of realism for the required task, with no difference noted between intermediate and advanced groups.

Participants in the expert group subjectively reported the arthroscopic view of the hip obtained following camera insertion was acceptably realistic. In contrast, greater trochanter palpation and location were felt to be unrealistic, in part, because of the lack of haptic feedback available for these steps.

### Content Validity

All intermediate and expert participants agreed the module required sufficient skills to be demonstrated, which were representative of the knowledge and intraoperative skills required for the procedure. Forty percent of participants reported the cannula insertion step to have only moderately acceptable levels of procedural relevance, because of the lack of active participation by the user required in this step of the module.

### Precision Score

**Fig 1:**
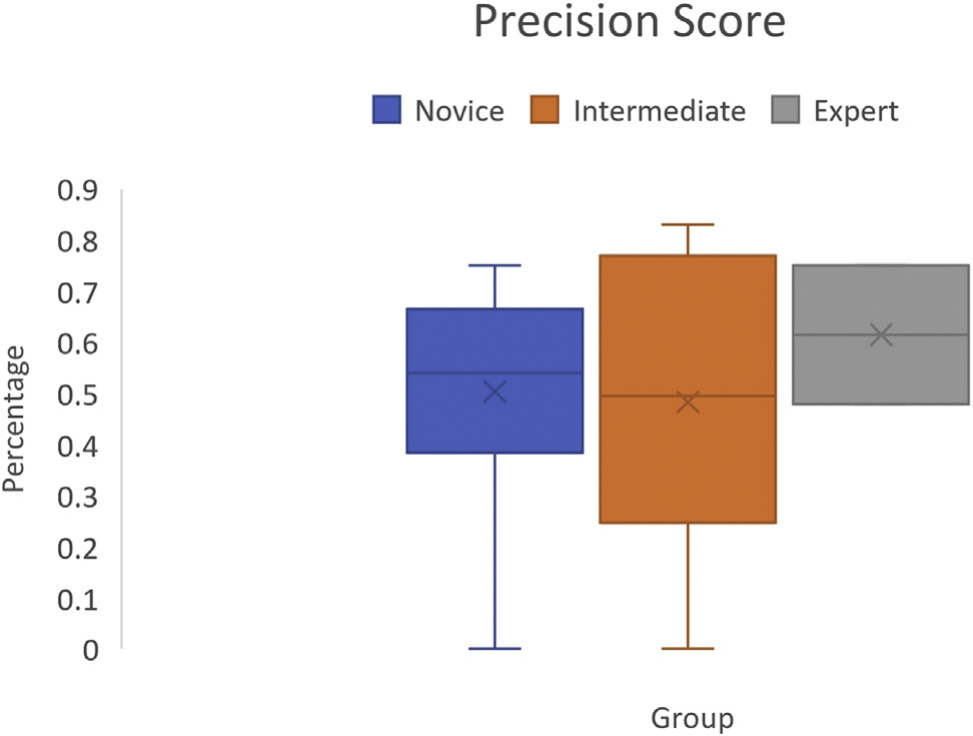
Precision Score shows no significant difference between groups.

Precision score was calculated by the simulator as a composite score from each individual metric collected and compiled by the software. The precision score outputted by the simulator noted no significant difference between scores achieved by participants in the expert, intermediate, and novice groups (62% ±19vs 55% ±22 vs 50% ±23,*P* = .15) (Fig 1).

Improvement between attempts was demonstrated in both novice (29% ±19 vs 50% ±23,*P* = .04) and intermediate (19% ±17vs 55% ±22,*P* = .003) groups. No improvement was seen in the expert group.

### Cam Lesion Location

**Fig 2:**
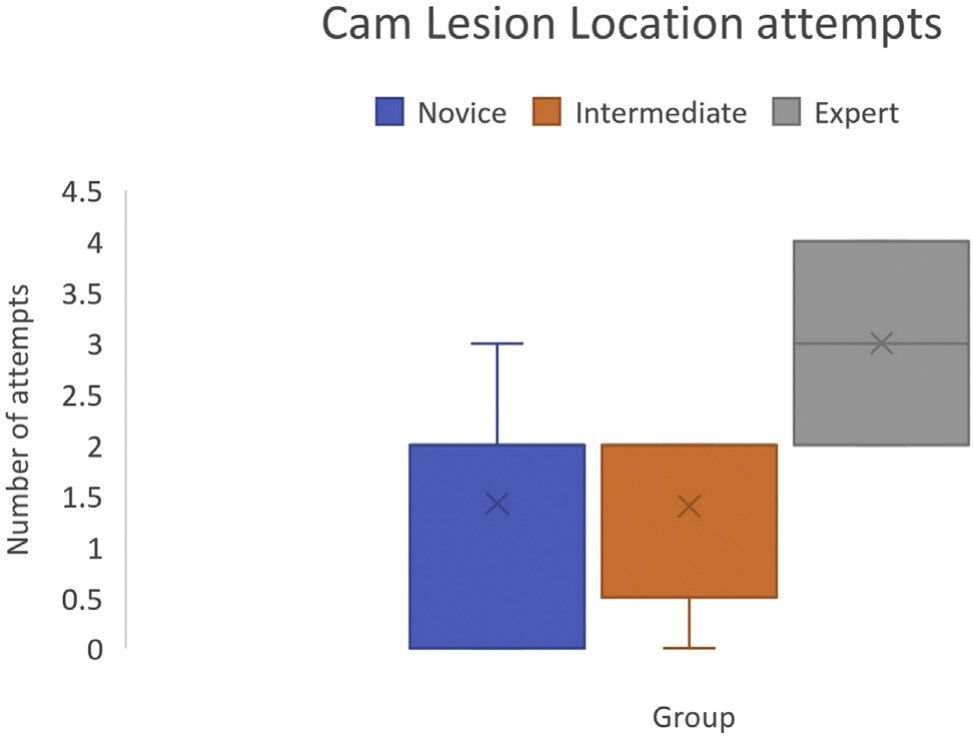
Cam lesion location attempts required, with no construct validity demonstrated.

Number of attempts to locate the cam lesion was not significantly different between groups (1.42 ±1.13 vs 1.4 ±0.89 vs 2 ±0,P=.5). Both novice and intermediate groups achieved the number of attempts required by experts (Fig. 2).

### Scope Strikes on Bone

**Fig 3:**
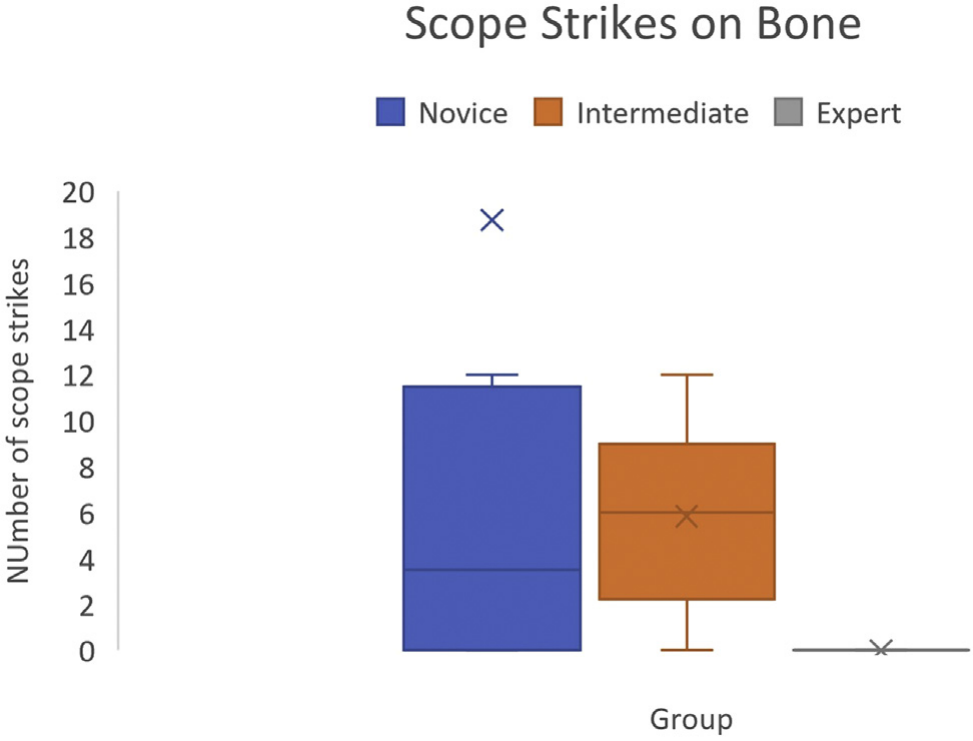
Scope strikes against bone demonstrated validity with experts having significantly fewer.

The mean number of scope strikes by experts was 0 (SD ±0). Novices had a mean of 5 strikes per attempt (SD ±5), with intermediatesincurring a mean of 5.8 strikes per attempt (SD ± 4.1). There was a significant difference between expert and novice groups (*P* = .01), and expert and intermediate groups (*P* = .002). No statistical difference found between novice and intermediate groups (*P* = .41) (Fig. 3).

### Healthy Bone Burred

**Fig 4:**
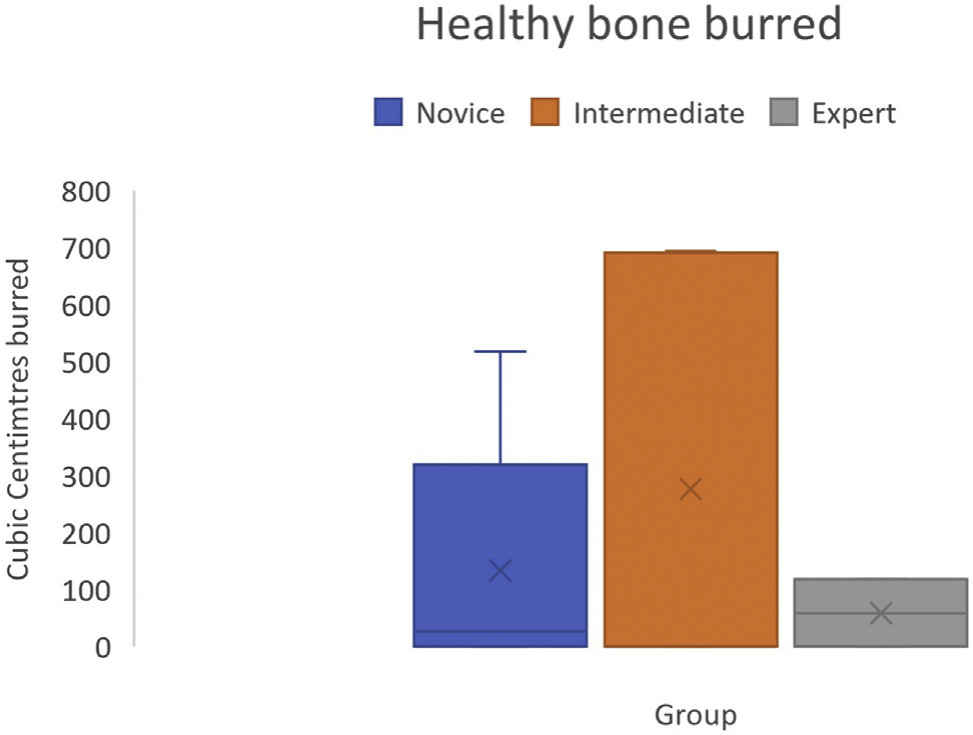
Healthy bone burred by cubic centimeters, with no difference noted between groups.

Volume of healthy bone burred was calculated in cubic centimeters. Mean volume burred by an expert was 59 cm^3^ (SD ±89). The mean total healthy volume burred by the novice group was 133 cm^3^ (SD ± 220) with the intermediate group burring a mean of 276 cm^3^ (SD ± 378). There was no significant difference found between expert and intermediate groups (*P* = .3) or expert and novice groups (*P* = .11). Difference in total volume burred between novice and intermediate groups was not statistically significant (*P* = .25) (Fig 4).

### Cam Lesion Burred

**Fig 5:**
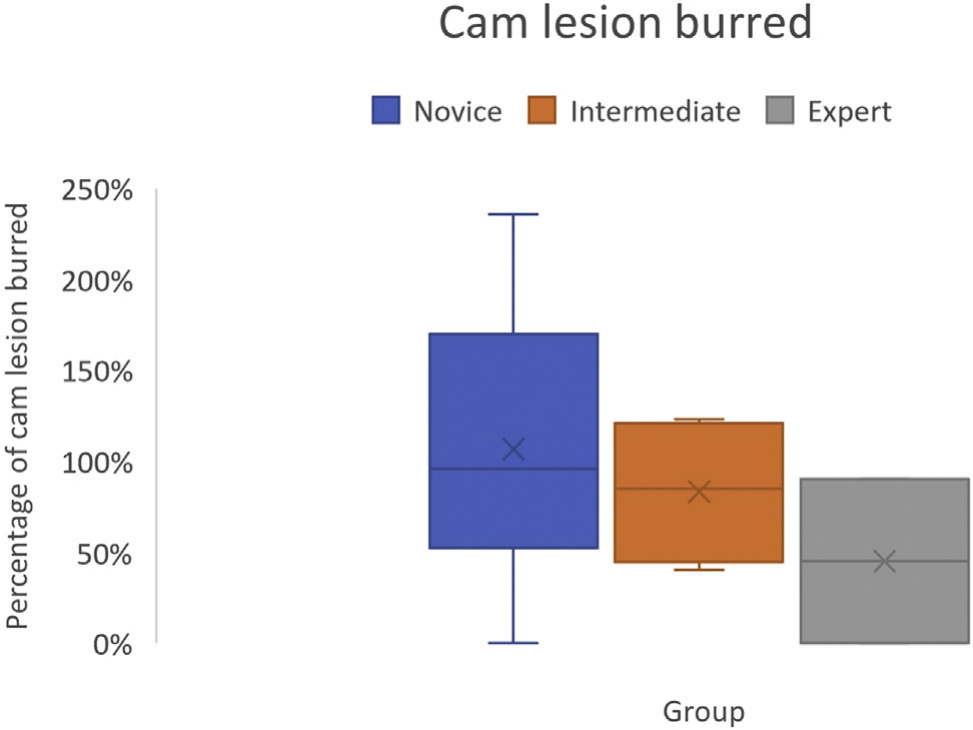
Percentage of cam lesion burred, with experts removing less, failing to achieve significance.

Volume of cam lesion was calculated as a percentage of bone burred. The mean percent of lesion burred was 45.5% (SD ±63) by experts, with 54.5% remaining following completion of the module. Intermediates burred a mean of 83% (SD ±40), with novices burring a mean of 106% (SD ±76)of the lesion. Amount of cam lesion burred was not statistically different across the three groups (*P* = .09), or between novice and intermediate groups (*P* = .11) (Fig 5).

### Objective Metrics

#### Time

Median time to completion by experts was 11 minutes (SD ±6). Intermediate group participants were slightly faster with a median of 9 minutes (SD ±6) to complete the module, although this did not reach significance (*P* = .32). Novices had a median of 14 minutes to completion (SD ± 4.3). There was no significant difference in time taken to completion between novice and intermediate groups (*P* = .22).

#### Impact on Patient Characteristics on Performance

**Fig. 6:**
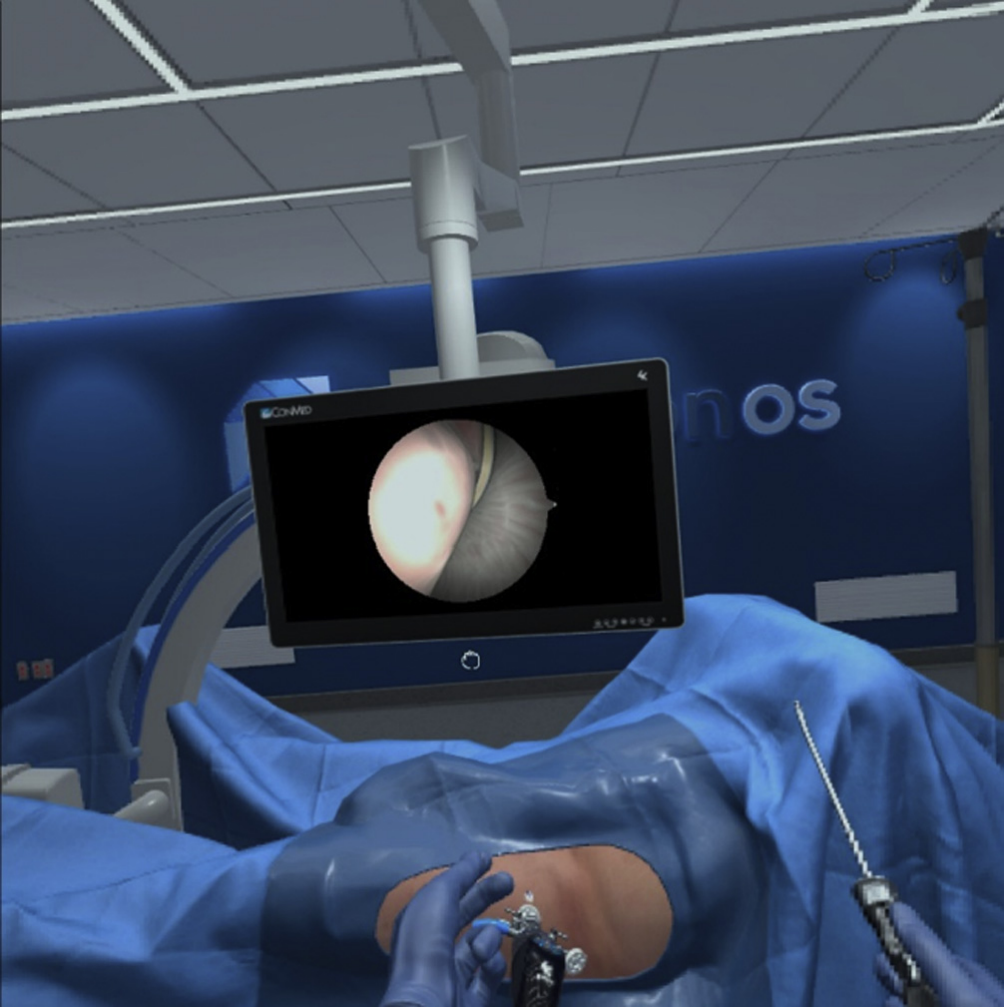
Initial arthroscopic view of left hip cam lesion.

**Fig 7:**
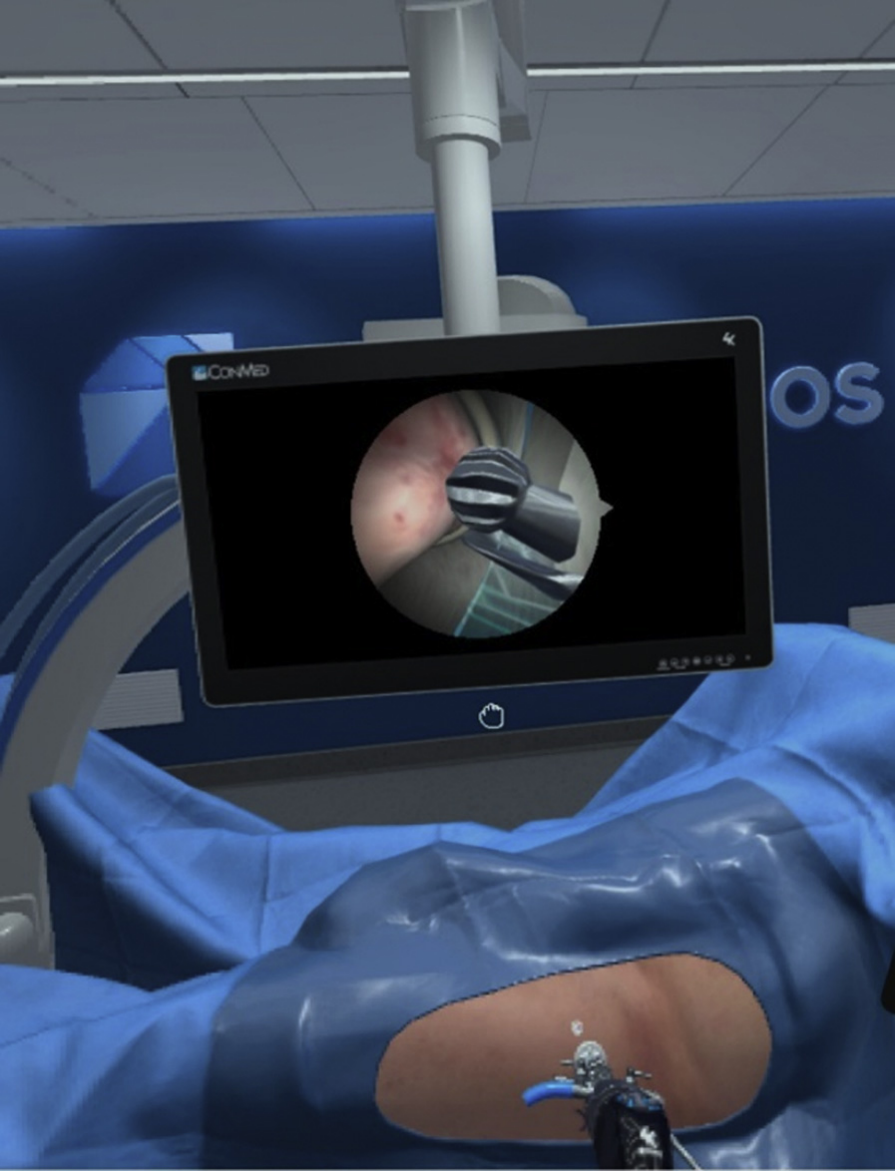
Left hip cam lesion with burr inserted and in view.

Previous virtual reality exposure does not appear to impact simulation or objective-derived metrics (*P* = .39). Only one left-handed participant was included in this study, and low number of participants with gaming experience precluded further analysis of these factors (Figs 6 and 7).

## Discussion

This study demonstrated acceptable levels of face and content validity for the Precision OS Cam Lesion hip arthroscopy procedure, with incomplete construct validity demonstrated. All participants reported the module had been sufficiently realistic to be an acceptable preparatory training tool for the procedure. Experts reported higher levels of realism for the arthroscopic picture generated than participants in either the novice or intermediate group. This may be due to the lack of familiarity of nonexperts with the in vivo procedure, a finding echoed in Alsalamah et al.^16^ Face validity is an integral first step in the development of a simulated procedure, as the tissues and instruments encountered virtually must be representative of in vivo procedures to be used for constructive surgical skill development.^16^

Several hip arthroscopy simulators are currently available. Face validity is most commonly used to identify initial validity in use of simulators,^14^^,^^17^ with sufficient levels of realistic components required to support simulator use in training inexperienced users to gain basic skill sets. Construct validity of hip arthroscopy simulators across individual modules from simulator models available^17^, ^18^, ^19^ have indicated the presence of both face and construct validity would support simulation-based training programs to be implemented.^14^ Similar to a validity study by Bartlett et al.,^20^ lack of tactile feedback was a limiting factor in the assessment component of the module. This is reflected in the performance of each of the groups, with the advanced group removing less than 50% of the lesion. The lack of haptic feedback, which this group would rely on in true arthroscopy, may account for this shortfall, with the group unable to perform in a manner reflective of their experience secondary to this.^2^^,^^3^^,^^14^^,^^17^^,^^19^^,^^21^

Four of the five steps in the module were accepted by participants of the group to be representative of the procedure, with skills required in the module to be reflective of those required in vivo. The cannula insertion step was only moderately acceptable to participants, with one expert reporting that its inclusion without the required active participation was not conducive to advancing surgical skill sets in surgical trainees. Virtual reality allows users to experience novel techniques in an environmentally safe, controlled environment.^3^ The most obvious advantage conferred by this is the opportunity for trainees to perform technically difficult procedures and maneuvers without compromising patient care and safety.^22^ Opportunities which promote the acceleration of surgical skills should be encouraged, with active and tactile feedback demonstrated to enhance learning compared to passive learning techniques.^23^

Scope strike on bone was the only metric analyzed, which could delineate between levels of experience. This is perhaps representative of instrument dexterity and safety within the expert group compared with the intermediate and novice groups, two key metrics in the validated ASSET score used to assess arthroscopic skills.^24^ Differences in the number of attempts required to locate the cam lesion and the amount of healthy and lesion bone burred were not significant between the groups, indicating only partial construct validity is available in this module. Time was also not a factor in delineating between levels of experience. The cannula insertion step was pretimed for 2 minutes to complete regardless of experience and should be considered a confounder in the use of time as a metric for experience in this module. Previous experience in arthroscopy of other joints did not appear to confer any advantage in this module. This is in contrast to previous studies, indicating a cross-over of arthroscopic skills.^3^

High levels of satisfaction were ubiquitous across the groups in this study. The module was reported by experts and intermediate groups to be more useful for new, inexperienced trainees and for clinicians with infrequent exposure to the procedure. Novices felt it would be useful for all users. Modules that provide basic procedural steps are often reported by users to be primarily useful for inexperienced trainees because of the perceived lack of skill expansion provided to experts.^16^^,^^25^

The “precision score” was not representative of the level of experience by the user, reflecting the lack of validity in the cumulative score from the individual metrics supplied. Further work is required to allow the software to accurately assess experience levels from inputted analysis.

### Limitations

This study has several limitations. The hip arthroscopy experience in the unit was generally low, resulting in limited recruitment numbers. Minimum numbers of participants were recruited on the basis of previous validity studies in arthroscopy to ensure adequate sampling; however, the study is limited, in that sample size was dictated by the number of surgeons available rather than by performing a priori power calculations. Surgical trainees in the intermediate group of participants reported relatively low levels of exposure to the procedure. Face validity is by nature subjective, and this must also be considered a limitation. Previous simulator exposure may have influenced subject responses, and this cannot be accounted for in the context of feedback given. The cannula insertion component of the module required a prerequisite amount of time to complete the step prior to moving on; a limitation to the utility of time to completion to delineate between experience levels.

### Conclusion

This hip arthroscopy simulator demonstrated acceptable face and content validity, with incomplete construct validity of simulator software metrics. Participants reported high levels of satisfaction with the module, highlighting that the addition of haptic feedback would be beneficial for improving procedural steps. Incorporation of tactile feedback to the modulator components would likely enable the software to accurately delineate between levels of experience.

## Supplementary Data

ICMJE author disclosure forms
